# Emergency department culture and the care of older patients with frailty: a focused ethnography

**DOI:** 10.1186/s12873-026-01550-7

**Published:** 2026-03-20

**Authors:** Dorien Venema, Laurien Kanis, Babiche E. J. M. Driesen, Nienke Bleijenberg, Lisette Schoonhoven, Marielle Emmelot-Vonk, Wietske H. W. Blom-Ham

**Affiliations:** 1 Research Group Proactive Care for Older People, University of Applied Sciences Utrecht, PO Box 12011, Utrecht, 3501 AA The Netherlands; 2 Department of General Practice and Nursing Science, Julius Center for Health Sciences and Primary Care, University Medical Center Utrecht, Utrecht University, PO Box 85500, Utrecht, 3508 GA The Netherlands; 3 Department of Surgical Specialties, Canisius Wilhelmina Hospital, Weg Door Jonkerbos 100, Nijmegen, 6532 SZ The Netherlands; 4 Department of Emergency Medicine, Meander Medical Center, Maatweg 3, Amersfoort, 3813 TZ The Netherlands; 5 Department of Geriatrics, University Medical Center Utrecht, Utrecht University, PO Box 85500, 3508 GA Utrecht, The Netherlands; 6 School of Health Sciences, Faculty of Environmental and Life Sciences, University of Southampton, University Road, Southampton, S017 1BJ UK; 7 Acute Care Network Central Netherlands, PO Box 85500, Utrecht, 3508 GA The Netherlands; 8 Wulverhorst 121, Montfoort, 3417TJ The Netherlands

**Keywords:** Older patients, Frailty, Culture, Emergency department, Emergency care, Ethnography, Qualitative research

## Abstract

**Background:**

Culture shapes care delivery, professional communication, and the development of trust between patients and providers. Culture encompasses identifiable elements, shared values and beliefs, and underlying assumptions that shape behaviour. The Emergency Department (ED) subculture arises from its complex, high-pressure focus on acute illness. ED professionals increasingly encounter older patients with frailty, whose complex but often non-urgent needs require more holistic, individualised care. However, the ED subculture may hinder the provision of appropriate geriatric emergency care. Despite its relevance, little is known of the influence of the ED subculture on the care for older patients. This study provides insight into the ED subculture in relation to the care of older patients with frailty by exploring its visible elements, shared values and beliefs, and underlying assumptions that shape behaviour.

**Methods:**

A qualitative, micro-ethnographic study was performed. Data were collected through topic-guided observations of ED professionals over twelve days, followed by interviews. Ethnographic methods were used to analyse the data, with iterative cycles of collection and analysis.

**Results:**

The themes ‘*Equality and professional solidarity as drivers of care delivery*’, and ‘*Responsibility and compassion in navigating the misalignment between emergency care and the needs of older patients with frailty*’ describe the ED subculture in relation to the care of older patients with frailty. The ED is highly structured, efficiency-driven, and professionals believe in equal care to all patients through responsibility and teamwork. Stabilising acute illness is prioritised during the initial assessment; other needs are considered once patients are stable or assertive. Professionals believe the ED is intended for acute problems; non-acute referrals of older patients are considered inappropriate. However, they value a strong moral responsibility, navigating between emergency care priorities and the needs of older adults with frailty.

**Conclusions:**

The ED subculture prioritises efficient, protocol-driven care and equal care for all patients, creating a challenge for delivering holistic, patient-centred care to older adults with frailty. Improving education and communication and integrating biopsychosocial principles into emergency care are essential. The ED subculture must be considered when developing and implementing future interventions to improve emergency care for older patients.

## Background

Culture plays a crucial role in healthcare organisations, shaping how care is delivered, how professionals communicate, and how trust is built between patients and healthcare providers [1, 2]. An organisation’s culture is visible at three levels [3]. The first level comprises identifiable elements, such as facilities, pathways, staffing, and dress codes. These elements include care behaviours and communication practices that are viewed as normal and acceptable. The second level comprises shared values and beliefs of a team, including arguments used to sustain current practice. The third level, and the most difficult to recognise, comprises underlying assumptions that shape behaviour [3]. Understanding these assumptions is crucial as they drive behaviour and decision-making in clinical practice [3]. If they remain hidden, changing behaviour can be difficult, and efforts to improve care often fail [2, 4].

Healthcare culture consists of multiple subcultures with distinct norms and practices; one of these subcultures is the emergency department (ED) [5]. The ED subculture has not been widely studied, and its levels, specifically the underlying assumptions that shape the behaviour of ED professionals, remain insufficiently understood. What is known about the ED subculture is that it is formed within a complex, high-pressure and stressful environment [6]. The identifiable elements reveal a system that is organised around triage and treatment of acute illness [6]. Professionals view the ED as the “gateway” to the hospital that needs to be available for the most urgent cases [6]. Important ED values include teamwork, punctuality, safety and reliability in care delivery and flexibility to adapt to high-pressure situations. ED professionals take pride in supporting patients at their most vulnerable and believe that comfort requires time, compassion, and clear communication [5]. An underlying assumption is that if it is not urgent, it can wait [5, 6]. This assumption is instilled during education and reinforced by the norms of the emergency care system [7].

Herein lies a fundamental paradox: EDs that are structured to prioritise rapid response to acute conditions are increasingly confronted with older patients whose presentations are complex yet not always urgent [7–9]. Older adults account for 30% of all ED admissions [10, 11]. Globally, they represent one of the fastest-growing population groups, estimated to reach 2 billion by 2050 [12, 13]. Due to a longer life expectancy, decline of physiological reserve across multiple systems, and the coexistence of multiple chronic diseases [10, 14], the risk of frailty also increases [15–21]. Frailty is defined as a reduced capacity across physical, cognitive, social, and psychological domains [15, 19, 22, 23]. ED presentations of older patients with frailty are often complex due to atypical complaints, co-morbidities and polypharmacy [8, 24, 25].

Meanwhile, older adults with frailty feel vulnerable during ED admission and tend to suppress their needs and place their trust in professionals [26–29]. Although older patients are often not explicit about their needs, they value holistic, individualised care and expect ED professionals to address their basic needs, such as clear communication, support in decision-making, comfort, and the provision of food and drinks [26–29]. ED professionals can minimise the impact of the ED admission by attending to the needs of older patients [26, 28, 30], however, the ED subculture may conflict with the nuanced and relational care these patients require [7, 31–34]. Consequently, ED professionals often lack the knowledge and capacity to provide appropriate geriatric care, in part because the complexity of care is not embedded in clinical guidelines and is not recognised within the ED culture as a patient group that requires specialised skills [6, 9, 35, 36].

While interventions exist to improve the quality of geriatric emergency care, their success may depend on how well they align with the ED’s subculture, the context in which the intervention will take place. Because the effectiveness of an intervention depends on context fit, understanding the context is essential to the success of a complex intervention [37, 38]. As a result, interventions aimed at improving emergency care for older adults may fall short if they do not account for the values, beliefs and underlying assumptions that form ED subculture [17, 39–41]. Research has provided insight into the care needs of older patients [26, 42, 43] and the challenges ED professionals face [9, 34, 36]. Although the role of ED subculture in care delivery has been investigated [5], little is known about the underlying assumptions that shape the behaviour of ED professionals. Furthermore, there is limited evidence on how the ED subculture influences care for older patients with frailty. Evidence on the core elements of the ED’s subculture helps optimise the development of interventions for older patients with frailty [2, 4, 6, 7]. This study aims to provide insight into the ED subculture in relation to the care of older patients with frailty by exploring its visible elements, shared values and beliefs, and the underlying assumptions that shape behaviour.

## Method

A micro-ethnographic approach, described by Holloway and Galvin, was used to explore the subculture of the ED in relation to the care for older patients with frailty [44]. This approach enables insight into the subculture, allowing values and beliefs to be described from a neutral perspective [5]. Micro-ethnography differs from traditional ethnography: it analyses “small” moments of human activity in natural settings, rather than focusing on larger-scale phenomena [45]. A small moment is an interaction or event that carries meaning. Observing these small moments is essential, as they provide insight into participant behaviour, an expression of the underlying values and beliefs within the ED subculture [45]. The principles of naturalistic inquiry guided the research process. These principles are aimed at interpreting participant behaviour within the natural context of the ED, without predefined assumptions [46].

### Setting and participants

The Dutch emergency care system is organised around general practitioners (GPs), emergency medical services (EMS) and EDs [47]. During office hours, patients can contact their GP for urgent medical needs or for a referral to the ED. After office hours, GP clinics can be contacted [47]. In case of a medical emergency, citizens can dial 112 for EMS. After EMS triage, a referral to the ED will be made if necessary [47, 48]. Patients can also access the ED by self-referral [48]. For this study, the setting concerned the ED of a large non-university teaching hospital providing secondary and tertiary care in the centre of the Netherlands. This ED admitted approximately 35.000 patients in 2024 (hospital finance department, personal communication, 2025) and provides emergency care to a broad, diverse patient population spanning all ages, acuity levels, and a wide range of medical, surgical, and trauma-related conditions.

In the Netherlands, patient safety in hospitals is organised through a national Safety Management System (VMS in Dutch), introduced between 2008 and 2012 as part of a nationwide patient safety programme supported by the Dutch Ministry of Health [49] This program includes 10 themes designed to reduce avoidable patient harm in hospitals by implementing structured, evidence-based safety practices. One theme is aimed at early identification of older adults with frailty, so risk can be anticipated and complications can be prevented during hospital care [39]. Implementation subsequently became a national standard for hospital care and ED care.

To explore the ED subculture, healthcare professionals providing direct patient care in the ED were purposively sampled and included emergency nurses, allied medical care professionals, nurse practitioners, physician assistants, emergency physicians, residents in specialist training, and non-training physicians. ED professionals were eligible for inclusion if they were 18 years or older and held a permanent contract in the ED. Exclusion criteria included nursing students and medical students in clinical rotation.

### Data collection

Data were collected from 14 February to 21 March 2023 through participatory observations, followed by in-depth interviews with the participants. Researcher LK (a registered nurse without ED experience and a novice nursing scientist) observed ED professionals over 12 shifts (day and evening) as they provided care to older patients with frailty. Older adults aged 70 or older were considered frail when they scored positively on at least one of the four Dutch Safety Management (VMS) Criteria for early identification of older adults with frailty: high risk of delirium, falls, malnutrition, or physical limitations (Table 1) [39].

**Table 1:** Dutch Safety Management System (VMS) criteria for early recognition of older adults with frailty

Patient ≥ 70 years old		
Domain	Screening question	Score
Delirium risk	▫ Do you have memory problems? ▫ In the past 24 h, have you needed help with self-care activities? ▫ Have you ever been confused or disoriented during a prior illness or hospitalisation?	0 = all no 1 = ≥ 1 yes
Fall risk	▫ Have you experienced one or more falls in the past 6 months?	0 = all no 1 = ≥ 1 yes
Malnutrition risk	▫ Have you experienced unintentional weight loss? ▫ Have you had decreased appetite in the last month? ▫ Have you used drink supplements or tube feeding in the past month?	0 = all no 1 = ≥ 1 yes
Physical impairment	▫ Do you need help with bathing or showering? ▫ Do you need help with dressing? ▫ Do you need help using the toilet? ▫ Do you use incontinence products? ▫ Do you need help transferring from bed to chair? ▫ Do you need help walking?	0 = < 2 yes 1 = ≥ 2 yes

VMS criteria derived from VMS veiligheidsprogramma kwetsbare ouderen [39].

The VMS criteria were applied upon the arrival of older patients at the ED. In cases where older patients needed immediate stabilisation, observation began before screening. Frailty was identified when the patient was stable; observations were excluded if the patient was retrospectively determined not to be frail. In practice, all included patients were classified as frail, so no cases were excluded.

Three weeks before data collection, researcher LK visited the ED to become familiar with the setting. Participants were informed verbally about the study’s purpose and received a digital information letter one week before data collection. Before each shift, participants provided informed consent. Demographic data, such as sex, age, and years of experience, were collected in Microsoft Excel (version 2509). The coordinating nurse notified the researcher when an older patient with frailty was admitted to the ED. The participant asked the patient for permission for the researcher to be present. Patients were informed that no personal data would be collected; therefore, written consent was not required. If a patient was unconscious, caregivers or the participant determined whether observation was ethically appropriate.

The participant observations were guided by a topic list (Table 2). The topic list was developed by researcher LK and the principal investigator WBH (a former ED nurse and professor in acute care), based on previous work on the needs of older patients with frailty in the ED [26]. To blend into the subculture, researcher LK wore a staff uniform, was present in the patient room and provided minor assistance. Notes were taken using thick description, focusing on the visible elements, interactions, emotions, and meaning to explore the subculture. In addition, short, unstructured interviews with participants were conducted to understand the values, beliefs, and underlying assumptions that guided the observed care. At the end of each shift, researcher LK documented the observation and interview notes in Microsoft Word (version 2509). For each observation shift, a transcript was created. The transcripts were not returned to the participants for member checking.

**Table 2:** Topic list used to guide the observations

Care needs of older patients with frailty	
What do older patients with frailty want?	To be seen; To feel welcome; To be recognised as a person rather than a disease.
What do older patients with frailty need?	Information and clarity during ED admission; To be approached with kindness and thoughtfulness; Awareness of their situation by the ED professional.
Attention to	Offering food and drinks; Opportunity for informal caregivers to be present.
**Care aspects**	
Environment and organisation	Context of the ED, including atmosphere, sounds, and temperature; Routines and processes; Involved disciplines.
Care delivery	Shared decision-making; Diagnostic tests; Medical and nursing assessments; Informal caregiver participation; Consideration of patient wishes; Responsiveness to patient emotions; Consideration of the patient’s personal situation; Consideration of patient vulnerability.
Interaction	Communication between ED professional and patient (verbal, non-verbal, barriers, patterns); Communication with informal caregivers; Attitude of the ED professionals toward the patient (and informal caregivers).

### Data-analyses

Data were collected during twelve observation shifts (4-day shifts and 8-evening shifts), over a five-week period. On two observation shifts, one day and one evening shift, no older patients with frailty were admitted to the ED. These two shifts occurred during exceptionally quiet shifts at the ED, with very few patients overall, and were not representative of typical patient flow in the ED. Therefore, a total of ten transcripts were analysed. The research team consisted of researcher LK, researcher DV (a former ED nurse and nursing scientist), and the principal investigator. Data were analysed following Holloway and Galvin’s six-step approach to ethnographic analysis (62), with interpretation of values, beliefs, and underlying assumptions from the researcher’s theoretical perspective. First, three transcripts were read repeatedly by researcher LK and the principal investigator to become familiar with the data. Second, meaningful words, phrases, and actions were independently coded and categorised. Third, patterns in behaviour and communication were identified and categorised into themes. After this, two additional rounds of observation days were performed, repeating steps one through three of the analysis. In the fourth step, themes were linked to the cultural context through research team discussions. In this step, researcher DV joined the analysis process to deepen the interpretation of categories and themes in relation to the research question. Themes were compared and discussed until consensus was reached on the possible underlying values. Following step five, the research team developed a conceptual framework to explain the relationships among themes. In the final step, researchers LK and DV wrote a detailed narrative, including quotes, observations, and interpretations of the subculture (63). The three levels of organisational culture: identifiable elements, values and beliefs and underlying assumptions, were used to guide the analysis [3].

The principles of naturalistic inquiry were followed from three perspectives [46]. The first, the observation perspective, is based on observed behaviours and actions. The second, the emic perspective, was captured through additional interviews, which provided insight into how participants assign meaning to their actions. Third, the etic perspective was demonstrated through researcher reflexivity, with constant reflection and team discussion on the influence of their own perspectives and interpretations on the subculture. Thematic saturation was achieved when no new themes emerged from the analysis, yielding comprehensive, rich data to answer the research question [50]. Data collection and analysis were performed iteratively. ATLAS.ti (version 25) was used to support the analysis.

### Trustworthiness

The criteria of trustworthiness by Lincoln and Guba were followed [46]. Credibility and confirmability were ensured through observations conducted at multiple points during the day and evening shifts over 5 weeks. Observation bias was minimised through regular debriefing sessions with the research team during data collection, in which the observer’s interpretations and potential influences of their positionality were critically examined and discussed. During data analysis, researcher triangulation was applied by involving multiple researchers. To promote dependability, the data collection, analysis, and methodology were thoroughly described. Thick descriptions were used to increase the transferability of the results.

## Results

Over ten observation days, seventeen ED professionals were observed: eleven female and six male participants, with a mean of 8 years of experience working in the ED. Thirty-seven distinct moments were observed, with a total length of 1060 (range 10–90) minutes. Each observation consisted of multiple “small” moments. Sometimes, a participant was observed at multiple moments involving the same older patient. At other times, multiple participants were involved in caring for a single older patient. Participant characteristics are displayed in Table 3. There were no dropouts.

**Table 3:** Participant characteristics

Participant	Profession	Sex*	Healthcare experience	ED experience#
1	Resident in specialist training	F	5	4
2	Resident in specialist training	M	3	1
3	Emergency nurse	F	8	3
4	Emergency nurse	F	15	11
5	Emergency Physician	M	13	4
6	Resident in specialist training	F	2	0.5
7	Emergency nurse	M	8	1
8	Emergency nurse	F	36	17
9	Emergency Physician	M	15	12
10	Emergency nurse	F	14	3
11	Allied medical care	M	6	3
12	Emergency nurse	F	27	23
13	Emergency nurse	M	6	6
14	Emergency nurse	F	26	15
15	Emergency nurse	F	41	20
16	Emergency nurse	F	16	10
17	Emergency nurse	F	24	8

*M Male; F Female^#^ In years

An observation about the visible elements showed that the ED appears to be a highly structured, functionally designed environment. When a patient arrived by ambulance, the coordinating ED nurse notified the ED nurse or an allied care professional responsible for the patient during the ED admission. This professional was the first to see the patient either in the ED hallway or after the patient had been brought into the room. After the handover from the EMS professional, the responsible ED nurse or allied medical care professional conducted the initial assessment using the ABCDE method. When a patient was in critical condition, an emergency physician was involved from the beginning. In other cases, the physician was informed after the initial assessment.

The corridors are white, and the patient rooms are brightly lit. Each room was private and centred around a narrow stretcher, with the head facing the door. The layout is minimalist, with medical equipment, such as monitors and infusion pumps, located at the head of each stretcher. One or two chairs are placed against the side wall, approximately 1,5 m from the patient. A desk with a computer is situated in a corner, either near the door or behind a stretcher. Constant stimuli mark the sensory atmosphere of the ED. Alarms and electronic beeps from medical devices are continuous, and healthcare professionals frequently enter and exit rooms. ED professionals all wear a white uniform. Nurses and allied medical care professionals wear short uniform jackets, while residents and physicians wear long jackets. Conversations in the corridor are audible within patient rooms, as doors are often left open and a curtain maintains privacy. Participants regularly receive phone calls and conduct brief conversations in the hallway or beside the patient.

After analysis of the data, the subculture of the ED in relation to the care of older patients with frailty can be described by two main themes that reflect the values, beliefs and underlying assumptions that shape behavior: *Equality and professional solidarity as drivers of care delivery*, and *Responsibility and compassion in navigating the misalignment between emergency care and the needs of older patients with frailty* (Table 4*).*

**Table 4:** Analysis framework

**Equality and professional solidarity as drivers of care delivery**
Striving for fair access to care
Shared responsibility and team-based efficiency
**Responsibility and compassion in navigating the misalignment between emergency care and the needs of older patients with frailty**
Clinical vigilance and moral responsibility
Compassion within time constraints
Shifting priorities

### Equality and professional solidarity as drivers care delivery 

#### Striving for fair access to care

Participants expressed a commitment to ensure accessible, fairly distributed care for all patients. Due to the high volume of patient admissions, rapid turnover and working efficiently are considered essential to ensure accessibility and availability of care. To achieve rapid turnover, participants identified acute illness using efficient methods and protocols. Participants believe that the ED is primarily intended for the care of acutely ill patients. In some situations, older patients were referred to the ED with what was considered by the participants as “simple or non-acute” problems that could have been managed elsewhere. This included older patients referred by a GP for diagnostic clarification, referral from a nursing home, or admission due to untenable home situations. The participants felt an implicit expectation that the ED would resolve these broader care issues.

*He [referring to the GP] assumes we’ll solve the problem and says it’s our responsibility*,* that really gets to me.* (Observation day 12, emergency nurse)

Participants were concerned when the ED was used for older patients with non-acute problems, and some even felt frustrated, feeling the ED was used unnecessarily. These feelings were sometimes verbalised in the presence of patients, reflecting the tension between interprofessional values and the realities of ED practice. The frustration experienced by ED professionals was sometimes expressed during discussions with the caregivers of older patients. When caregivers expected the patient to be admitted, ED professionals explained the purpose of the ED and the hospital policy.

*I would like to prepare you for this*,* since this is not how things work here.* (Observation day 12, emergency nurse)

#### Shared responsibility and team-based efficiency

Participants felt a collective responsibility towards their colleagues, aiming to complete tasks before shift changes to avoid passing on unfinished work. Strong interpersonal relationships and mutual respect within the team reinforced this sense of professional solidarity, which was further strengthened through humour and the sharing of personal experiences.

To maintain flow and efficiency, diagnostic procedures began immediately upon the older patient’s arrival, with a focus on the patient’s physical complaints. Older patients were assessed using standardised protocols, with a focus on identifying the acute illness and stabilising the patient. During the initial assessment, time, efficiency and clarity were a priority. Questions about individual needs were rarely asked, and treatment plans were communicated without verifying patients’ understanding.

*It is busy here [in the ED]*,* and we have limited time to do all the medical examinations*,* so that needs to be done to come first.* (Observation day 3, emergency nurse)

When multiple participants cared for the same older patient together, procedures were performed simultaneously. Participants appeared to have an intuitive understanding of their role within the care process, working as a team and performing their tasks almost automatically. Teamwork was evident in how tasks were coordinated. For example, one participant measured the patient’s blood pressure while a colleague drew blood from the other arm, and another simultaneously performed a COVID-19 test by swabbing the patient’s nose. Helping each other stabilise the patient quickly and efficiently showed a shared responsibility. During these procedures, older patients were informed that “it only takes a moment”.

*This will be unpleasant for a moment*,* but once it’s done*,* I’ll leave you in peace. I don’t like bothering you like this.* (Observation day 9, emergency nurse)

Older patients sometimes struggled to comprehend the information provided by participants due to hearing impairments or environmental distractions. In some cases, older patients arrived at the ED without their hearing aids, whereas in others, patients with hearing aids were asked to remove them during routine procedures, such as temperature measurement. In both situations, questions or explanations were sometimes delivered in a manner that patients could not hear or fully understand. Older patients appeared easily distracted by the surrounding environment, including room alarms, phone calls, and conversations among other healthcare professionals present. Meanwhile, the healthcare professional at the bedside attempted to communicate with the patient, who was often distracted and unresponsive to the questions posed. When patients failed to follow instructions, caregivers, initially asked to wait on the side and allow the professionals to do their work, were invited to assist. In other situations, participants frequently took over tasks from older patients during diagnostic procedures to accelerate the workflow. For example, undressing the patient when they were unable to do so quickly on their own. During these moments, privacy was not always prioritised, leaving patients’ doors open and allowing visibility from the hallway.

During the initial assessment, participants paid minimal attention to psychosocial care. Sometimes patients wanted to share their personal stories and ask questions. These questions were often not addressed because diagnosis and treatment came first. Participants explicitly stated there was no time to elaborate at the moment, indicating they would return to them later. When an older patient asked when they could go home, the participant made clear there were other priorities.

*We need to start the treatment first; going home is something to worry about later.* (Observation day 1, resident in specialist training)

### Responsibility and compassion in navigating misalignment between emergency care and the needs of older patients with frailty

#### Clinical vigilance and moral responsibility

ED professionals thoroughly investigated patients’ physical symptoms, even when the urgency of the situation was unclear. Participants noted that the slower pace of older patients does not align with the ED’s fixed routines. Although they experienced the misalignment between emergency care and the needs of older patients, deviating from established routines proved difficult in practice. However, participants felt a strong moral responsibility to provide high-quality, patient-tailored care for older patients with frailty, despite the fast-paced nature of the ED routines.

*You are now in the emergency department*,* you are quite sick*,* so this cannot wait. We want to look thoroughly at what is going on. *(Observation day 12, Resident in specialist training)

Participants suggested that older patients would benefit more from a calm, low-stimulus environment. They feel that the high level of sensory stimuli, the constant presence of people and noise, and the ED’s physical layout are often not optimal for older patients. Although participants felt that ED admission might be harmful for older patients, they were sometimes hesitant to identify such situations and to adjust their care to align with patients’ needs.

*An ambulance ride*,* then you’ve already got that delirium*,* this [admission aimed at ruling out delirium] is the greatest nonsense.* (Observation day 1, emergency nurse)

#### Compassion within time constraints

ED professionals acknowledged that ED admission was stressful and overwhelming for older patients. To reassure patients that they were being cared for, participants encouraged them to “trust them” and to let the procedures unfold.

*We are doing everything very quickly*,* and it may feel impersonal*,* but this is the best way to help you. *(Observation day 8, emergency nurse)

Participants showed compassion to older patients through small gestures during diagnostic procedures. For example, placing a hand on the patient’s shoulder, gently holding their hand or providing pre-warmed blankets. Participants tried to lift patients’ spirits and relieve tension by using humour to make contact with patients and their caregivers. Brief moments of attention, expressed through short remarks to the patient, suggested that patients were seen and acknowledged. These actions conveyed compassion and attentiveness within the time constraints of the ED.

What *a tough situation*,* huh? Things haven’t been going your way.* (Observation day 10, emergency nurse)

#### Shifting priorities

Once the initial assessment was finalised and the patient was stabilised, participants shifted their priorities to providing comfort and personal attention. Participants explained the treatment calmly to the patient, involving caregivers in care discussions, and showed genuine interest in the patients’ home and social context. Emotional well-being was addressed through questions about feelings and concerns.

At this stage, basic needs were also attended to; patients were helped to sit upright, offered food and drink, and assisted with toileting. Participants cleared space for caregivers to sit beside the patient. Upon leaving the room, participants reassured patients and families that they could call for help at any time.

ED professionals were continually seeking a balance between efficiency and the needs of older patients with frailty to fulfill their moral obligations. This quest was reflected in comments exchanged among participants, which often expressed reluctance or frustration about tasks they did not want to perform but felt obliged to carry out. For example, when they felt the routine care did not meet the patient’s needs, or when they disagreed with the GP’s decision to refer the patient to the ED.

*What on earth are we doing? It feels contradictory to carry out all these examinations when the patient does not want them at all.* (Observation day 1, emergency nurse)

Even when participants felt the need to shift their priorities and adjust care to the patient’s needs, deviating from routine care was difficult for ED professionals. Diagnostic procedures were often routinely performed to either rule out potential issues or to ensure nothing was wrong.

*It’s proper to draw a quick blood sample* (Observation day 1, emergency physician)

When an older patient was assertive about their needs and preferences, it was easier for participants to shift their priorities. ED professionals would acquire information about patients’ wishes when patients repeatedly indicated a preference for something other than the standardised ED treatment. In these situations, patients’ wishes were attended to when possible, or the reasons for not doing so were explained. When a patient was considered confused and unable to understand what was happening, their preferences were less frequently addressed. In such situations, ED professionals sometimes interrupted patients to indicate that the assessment or procedure needed to continue.

*I need to interrupt you for a moment; you’re talking a lot*,* but we still have a few things to do.* (Observation day 9, emergency nurse)

## Discussion

The results show the ED subculture in relation to the care needs of older patients with frailty. The ED environment is highly structured and functional. ED professionals believe in equality of care for all patients. To ensure equality of care, ED professionals work efficiently and share responsibility. Professional solidarity and team-based efficiency are highly valued. The ED subculture prioritises identifying acute illness during the initial assessment. ED professionals believe that other needs have to wait until the patient is stable. ED professionals can address other needs during the initial assessment when patients are assertive and able to verbalise their needs. Professionals believe the ED is primarily intended for the care of acutely ill patients. Therefore, when older patients are referred to the ED with what they perceive as non-acute problems, they feel the ED is used inappropriately. However, ED professionals value a strong moral responsibility and compassion in navigating the misalignment between emergency care and the needs of older patients. These values guide their efforts to balance the priorities in emergency care with the specific needs of older patients with frailty.

The strong priority to identify acute illness by using protocols during initial assessment aligns with existing descriptions of the ED subculture [5, 6]. The ED subculture is characterised by rapid decision-making and a biomedical approach, with a focus on life-saving actions [6, 51]. These priorities originate from military medicine, where triage and protocol-based work were developed for the rapid treatment of trauma patients [52, 53]. This approach was later adopted by modern emergency medicine [52]. The difficulty of addressing the needs of older patients with frailty in the subculture of the ED can be attributed to the deeply rooted biomedical approach in ED care [34]. In the biomedical approach, diseases and health are viewed as biological and physiological processes that can be understood, diagnosed, and treated through medical science and techniques [54]. Integrating the biopsychosocial approach can help ED professionals address the complex care needs of older patients [55]. The biopsychosocial approach focuses on holistic, patient-centred care by addressing problems across the biological, psychological, and social domains [21, 54, 55].

Our findings show that ED professionals are aware of the misalignment between emergency care and the needs of older patients. Yet, they rarely deviate from established protocols, as these routines are perceived as “the way we always do things”, and deviation is seen as disruptive to the process. These protocols and procedures are the identifiable elements of the organisation’s culture [3]. These elements are reinforced by the shared value that rapid and efficient action is the essence of good emergency care, with the primary goal of saving lives [31, 56, 57]. Beneath this value lies a deep-rooted assumption that strict adherence to protocols and efficiency can save lives and is therefore followed without question [6, 31, 56]. This could explain why professionals conform to the existing ED subculture and find it challenging to adapt to care needs that are not directly lifesaving. To help ED professionals adjust their care for older patients, it is necessary to provide training and education focused on geriatric care [9, 34, 35, 58].

ED professionals were able to shift their priorities when patients were assertive and verbalised their needs. Pavedahl et al. (2022) show that emergency nurses were able to address fundamental care needs when communicating with patients [59]. Communication was essential to establish a nurse-patient relationship [59]. They found that a nurse-patient relationship was often established after the initial assessment. When this relationship was established earlier on, psychosocial and relational needs were addressed [59]. Little is known about the influence of patient assertiveness in the ED. However, we know that greater patient assertiveness is associated with improved healthcare outcomes in general [60], and communication influences patient outcomes [61]. Therefore, communication could be the key to helping ED professionals attend to the needs of older patients.

We observed a challenge for ED professionals to address the basic needs of older patients with frailty due to the ED subculture. This challenge may relate to the difficulty of integrating holistic, patient-centred care in the ED [59]. Basic needs are an essential part of fundamental care [62]. Fundamental care encompasses the care that patients require, regardless of their clinical condition or the setting in which they receive care [62]. It refers to the physical, psychological, and relational aspects of care that must be addressed to ensure a patient’s well-being [62, 63]. These include, for example, communication, information, involvement in care decisions, personal hygiene and nutrition [63, 64]. In our study, these care needs were addressed mainly after the patient was stabilised. This reflects how emergency care systems tend to undervalue fundamental care, prioritising protocolised and acute, illness-focused tasks over personalised care [65]. Emergency nurses often report that fundamental care receives little emphasis, is not clearly defined as part of their role, and is therefore deprioritised amid competing demands [60, 65]. Consistent with this, our findings show that identifying and managing acute illness takes precedence over all other tasks.

The feeling that the ED is inappropriately used when older patients with non-urgent complaints are admitted is also reflected in other studies [6, 56]. ED professionals often care for patients who do not require urgent care and are perceived as falling outside the intended scope of emergency services [56]. Because of this experience, emergency nurses believe that the ED hinders the provision of good care for older patients [7, 9]. They feel that the ED is not the right place for older patients, as the environment is poorly suited to them and their needs are not prioritised [9, 66]. The perceived unnecessary admissions cause frustration and can lead to moral distress among ED professionals [34, 67]. Despite this frustration, they feel a strong responsibility to provide the care they deem necessary for older patients [7, 34, 67]. This corresponds to the strong moral responsibility of ED professionals in our study.

Although our study focuses on older patients with frailty, comparable findings have been reported in other patient groups whose needs extend beyond the acute biomedical scope of the ED. For example, people presenting in mental health crises frequently describe the ED as poorly suited to their needs [68, 69]. ED professionals similarly identify training and protocol gaps that limit the care provided to patients with mental illness [70]. Patients with medically unexplained symptoms or non-urgent presentations often do not fit the ED’s protocolised priorities, which contributes to frustration, over-investigation, and perceptions of “inappropriate use,” reflecting a mismatch between biomedical imperatives and person-centred needs [71]. Individuals with complex social needs (e.g., homelessness, overlapping equity-deserving identities) more often report feeling judged, disrespected and unable to influence their care, underscoring how structural and cultural features of emergency care can impede fundamental, relational care across different patient groups [72].

### Strengths and limitations

A key strength of this study lies in its micro-ethnographic approach, which provided a unique and detailed insight into the subculture of the ED. This approach, combined with a diverse research team, enhanced reflexivity and provided nuanced insight into the ED subculture. The research team included members with and without ED experience. Because researcher LK had no experience with ED, the observations could be approached without preexisting assumptions, while prior knowledge of hospital care facilitated understanding of procedures and context. The authenticity of observations was enhanced by wearing the same uniform as the ED staff, participating in breaks, and sharing practical experiences. Being a part of the ED team helped build trust and facilitated access to natural team interactions. To avoid disrupting patient care, notes were taken after observations rather than during clinical interactions, preserving the integrity of the observed dynamics.

However, the study also had some limitations. Most observations involved nurses, as they are typically the first point of contact for patients. Emergency physicians were observed less frequently because they were involved later in the care process. This may have influenced the representation of interprofessional dynamics. Furthermore, the study was conducted in a single ED setting, and cultural and organisational differences in other hospitals may lead to variations in practice. To mitigate this, findings were discussed with experts to ensure broader interpretability. In addition, neither the ED professionals nor the older patients with frailty were blinded to the presence of the researcher during observations. In a micro-ethnographic design, the visible presence of the researcher in the field is inherent to the methodology and cannot be concealed. Although this visibility may have influenced behaviour to some extent, prolonged engagement and repeated observations helped reduce the likelihood that this had a major impact on the findings.

### Implications for emergency practice

The findings of this study underscore the need for greater awareness among ED professionals of the influence of the ED culture on the care for older patients with frailty. Recognising these cultural dynamics can help professionals adapt their practice and prioritise holistic patient-centred care. To empower ED professionals to adapt, it is essential to include geriatric and fundamental care in training and education. To improve interactions in emergency practice, training and education must adopt a biopsychosocial approach that supports both effective management of acute conditions and holistic, patient-centred assessment of the needs of older patients with frailty.

### Implications for further research

Future research should focus on developing and implementing interventions that embed holistic, patient-centred care for older patients in the specific context of emergency care. In addition, more research is needed to examine broader systemic changes that can be achieved across emergency care settings. This includes improving training, education and communication. Introducing integrated, biopsychosocial care early in education will improve patient-centred care delivery to older patients with frailty. Such initiatives should actively involve both ED professionals and older patients, as these changes directly affect both groups. Collaborative design processes can ensure that proposed changes are practical, acceptable, and aligned with patient values and clinical realities. In addition, future research should further explore how the underlying assumptions of ED professionals, shaped not only by their professional norms but also by their human, moral, and ideological views, influence their professional identity. Examining them may help clarify how deeply embedded beliefs reinforce or challenge the existing ED subculture.

## Conclusion

This study provides a unique insight into the ED subculture and its influence on care provided to older patients with frailty. At the ED, team practices are shaped by prioritising efficient, protocol-driven care and equality of care for all patients. This creates challenges in delivering holistic, patient-centred care. While ED professionals express a strong moral responsibility towards older patients with frailty, structural and cultural constraints limit their ability to address fundamental care needs adequately. These insights underscore the importance of improving education and communication and integrating biopsychosocial principles into emergency care settings. Future research should focus on developing interventions to support ED professionals in providing holistic, patient-centred care to older patients with frailty. In the development and implementation of these interventions, the influence of the underlying assumption of ED professionals on the ED subculture must be considered.

## Data Availability

The data that support the findings of this study are available from the corresponding author upon reasonable request. Interview data and analysis are in Dutch. The data are not publicly available because of privacy and ethical restrictions.
